# Environmental Risk Factors for Schizophrenia and Bipolar Disorder and Their Relationship to Genetic Risk: Current Knowledge and Future Directions

**DOI:** 10.3389/fgene.2021.686666

**Published:** 2021-06-28

**Authors:** Natassia Robinson, Sarah E. Bergen

**Affiliations:** Department of Medical Epidemiology and Biostatistics, Karolinska Institutet, Stockholm, Sweden

**Keywords:** schizophrenia, bipolar disorder, risk factors, gene-environment interaction, marijuana use, migration, pregnancy complications, adverse childhood experiences

## Abstract

Schizophrenia (SZ) and bipolar disorder (BD) are severe psychiatric disorders which result from complex interplay between genetic and environmental factors. It is well-established that they are highly heritable disorders, and considerable progress has been made identifying their shared and distinct genetic risk factors. However, the 15–40% of risk that is derived from environmental sources is less definitively known. Environmental factors that have been repeatedly investigated and often associated with SZ include: obstetric complications, infections, winter or spring birth, migration, urban living, childhood adversity, and cannabis use. There is evidence that childhood adversity and some types of infections are also associated with BD. Evidence for other risk factors in BD is weaker due to fewer studies and often smaller sample sizes. Relatively few environmental exposures have ever been examined for SZ or BD, and additional ones likely remain to be discovered. A complete picture of how genetic and environmental risk factors confer risk for these disorders requires an understanding of how they interact. Early gene-by-environment interaction studies for both SZ and BD often involved candidate genes and were underpowered. Larger samples with genome-wide data and polygenic risk scores now offer enhanced prospects to reveal genetic interactions with environmental exposures that contribute to risk for these disorders. Overall, although some environmental risk factors have been identified for SZ, few have been for BD, and the extent to which these account for the total risk from environmental sources remains unknown. For both disorders, interactions between genetic and environmental risk factors are also not well understood and merit further investigation. Questions remain regarding the mechanisms by which risk factors exert their effects, and the ways in which environmental factors differ by sex. Concurrent investigations of environmental and genetic risk factors in SZ and BD are needed as we work toward a more comprehensive understanding of the ways in which these disorders arise.

## Introduction

Schizophrenia (SZ) and bipolar disorder (BD) are severe psychiatric disorders affecting ∼0.7 and ∼1.0% of the population, respectively (McGrath et al., 2008; Merikangas et al., 2011). Both have a typical onset during late teenage years or in the early 20s and are associated with substantial morbidity and premature mortality (Lambert et al., 2003; Carney and Jones, 2006; Kilbourne et al., 2009). Furthermore, the clinical presentations of these disorders can overlap. Delusions and hallucinations occur in nearly all cases of SZ but also in about half of cases of BD (Coryell et al., 2001; American Psychiatric Association, 2013). Depressed mood, an essential feature of BD, can be hard to distinguish from the negative symptoms of SZ. Both BD and SZ often present with symptoms of grandiosity or paranoia.

Considering their similarities, it is not surprising that the etiological sources for these disorders have been compared, and the genetic foundations are substantially shared (Lichtenstein et al., 2009; Bulik-Sullivan et al., 2015). Risk for both disorders stems primarily from genetic sources, with heritability estimates from twin and family studies ranging from 64–81% for SZ (Sullivan et al., 2003; Lichtenstein et al., 2009) and 60–85% for BD (Smoller and Finn, 2003). Although there is significant shared genetic risk between several psychiatric disorders, the strongest correlation is between SZ and BD (*r*_g_ = 0.60–0.68) (Lichtenstein et al., 2009; Lee et al., 2013; Pettersson et al., 2016; Brainstorm Consortium, 2018). Great strides have been made in revealing the specific genetic loci associated with these disorders. Genome-wide association studies (GWAS) have now discovered 270 single nucleotide polymorphisms (SNPs) conferring risk for SZ (Ripke et al., 2020) and 64 for BD (Mullins et al., 2020), with some overlapping associations. However, many of these common genetic variants are not shared between these disorders, and other forms of genetic variation, such as copy number variants (CNVs) (Malhotra and Sebat, 2012; Marshall et al., 2017) and rare variants identified through sequencing (Purcell et al., 2014; Goes et al., 2016), appear to play a much larger role in the genetic architecture of SZ than BD.

Discovery of the environmental sources of risk has not kept pace with the dramatic advances in understanding the genetic underpinnings for SZ and BD, and the extent to which genetic sharing of risk may be mirrored for environmental factors is not well known. Speculation and investigation regarding the risk for SZ and BD which is derived from environmental factors stretches back for decades, with particular focus on exposures early in life, such as: winter/spring birth, obstetric complications (OCs), infections, and adverse childhood experiences (ACEs). However, urban living, migration, and cannabis use are usually later exposures which have also been the focus of intense interest in relation to SZ and, to a lesser extent, BD (Table 1). Of the numerous studies of environmental risk factors for SZ, some have yielded reproducible findings. While for BD, there have been fewer studies, usually with smaller sample sizes and inconsistent findings.

**TABLE 1:** Summary of existing evidence for environmental exposures and genetic risk for BD and SZ.

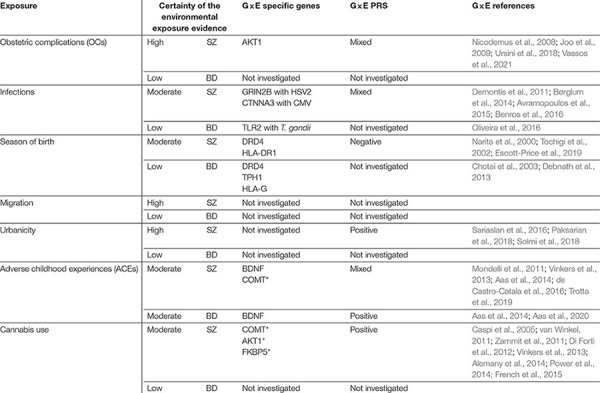

*Outcome was psychosis. G×E references from candidate gene studies and PRS. Low, very few studies with no consistent findings/inconclusive; Moderate, supported by some studies (may be small samples and mixed results); High, replicated results, consistent findings, large studies conducted; Positive, studies support of G×E interactions; Negative, studies do not support G×E interactions; Mixed, findings are inconsistent; HSV2, herpes simplex virus-2 in utero; CMV, cytomegalovirus – in utero; GRIN2B, glutamate ionotropic receptor NMDA type subunit 2B; CTNNA3, catenin alpha 3; BDNF, brain-derived neurotrophic factor; COMT, catechol-O-methyltransferase; AKT1, AKT serine/threonine kinase 1; FKBP5, FKBP prolyl isomerase 5; HLA-DR1, major histocompatibility complex, class II, DR beta 1; DRD4, dopamine D4 receptor gene.

Furthermore, risk from genetic and environmental sources may not contribute in an additive manner, since the impact of an environmental exposure may depend on the genetic makeup of the person experiencing it. These gene-by-environment (G×E) interactions partly explain why only some people who experience environmental exposures associated with SZ or BD actually develop these disorders, and identifying the genetic risk factors conferring vulnerability to specific environmental insults may open opportunities for prevention for these devastating disorders. Additionally, providing a comprehensive etiological picture of these disorders necessitates an understanding of the relationship between the genetic and environmental factors which contribute to the shared and disorder-specific symptoms.

**FIGURE 1:**
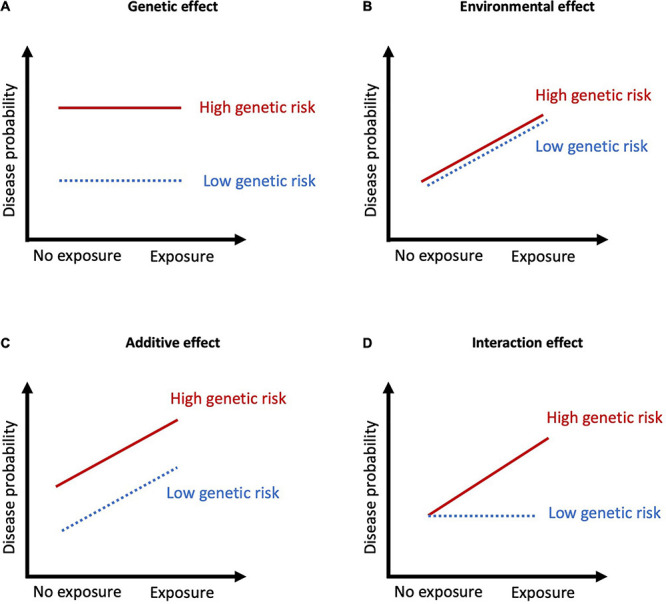
Summary of the types of G×E interactions. **(A)** Genetic effect with no exposure effects, meaning the variation in disease probability is stable across a range of environments; **(B)** Environmental effect with no genetic effects, meaning the variation in disease probability is stable across different genotypes; **(C)** Additive effects occur when disease probability results from the addition of the genetic and environmental factors. The exposure results in the same degree of increase in disease probability; **(D)** Interaction effects when genetic risk determines sensitivity to environmental factors. When there is no exposure, disease probability is low regardless of genetic risk, but in the presence of an exposure, those with high genetic risk have markedly increased probability of disease.

Examining interaction effects requires even larger sample sizes than for genes or environmental factors alone, which poses an additional challenge when investigating psychiatric disorders which affect a small proportion of the population. Indeed, the early G×E studies involving candidate genes were often underpowered, prone to publication bias, and usually lacked replication (Duncan and Keller, 2011). Greater certainty in the genetic markers conferring risk for SZ and BD and larger samples have improved these studies over time. Additionally, methodological advancements have provided an alternative approach to examining individual SNPs or genes. Polygenic risk scores (PRS) index genomic risk for a disease or trait by aggregating the effects of SNPs across the genome weighted by their effect size from a discovery GWAS in a separate sample (International Schizophrenia, Consortium, Purcell et al., 2009). PRS are individual measures of genomic risk which are continuous and normally distributed in the population, and they can be used to test for interactions with environmental measures. Several studies using either individual or aggregated genetic markers have investigated the relationship between environmental exposures and genetic risk for BD and SZ (Figure 1).

This review presents a thorough examination of the current evidence for environmental exposures which have been studied in relation to SZ and BD, and when available, their reported interactions with different forms of genetic risk. We also evaluate emerging environmental risk factors and conclude by addressing the current knowledge gaps and future directions for this field of research.

## Environmental Risk Factors

Although SZ and BD arise predominantly through genetic risk, ∼15–40% of risk for both is derived from environmental factors (Smoller and Finn, 2003; Sullivan et al., 2003). There have been numerous studies of environmental risk factors for SZ which have yielded some reproducible findings. The same cannot be said for BD, for which there have been fewer studies, usually with smaller sample sizes, inconsistent findings, and no firm conclusions. Although the environmental risk factors for SZ and BD have been extensively reviewed separately (Brown, 2011; Marangoni et al., 2016; Belbasis et al., 2018), there has been little research concomitantly examining both disorders.

Previous research on environmental risk factors has predominantly focused on OCs, infections, season of birth, migration, urbanicity, cannabis use or ACEs (Table 1). Each of these key exposures and their relation to genetic factors will be discussed further.

### Obstetric Complications

In systematic reviews, complications during pregnancy or delivery, or abnormal fetal growth and development have been associated with later development of SZ in offspring (Cannon M. et al., 2002). However, associations between distinct OCs and SZ are inconsistent across studies (Cannon M. et al., 2002) and evidence for associations with BD is weak (Scott et al., 2006). Oxygen insufficiency is a common feature across several consistently associated OCs and there is support for fetal hypoxia as an underlying mechanism from both observational studies (Dalman et al., 2001; Cannon T. D. et al., 2002; Byrne et al., 2007) and animal model studies (Boksa, 2004). For example, perinatal asphyxia has been associated with over 4× increased odds of SZ after controlling for other OCs, maternal psychosis, and socioeconomic status in a Swedish study (Dalman et al., 2001). Similarly, indicators of hypoxia, along with prematurity, were also associated with SZ in data from the Danish registries (Byrne et al., 2007). In the United States Collaborative Perinatal Project, individuals with 3+ hypoxia-related OCs were over 5× more likely to develop SZ compared to those with no hypoxia-related OCs (Cannon et al., 2000). Furthermore, the effects of fetal hypoxia are exacerbated in those born small for gestational age (Cannon T. D. et al., 2002), which indicates interactions between individual OCs, complicating interpretation of their effects.

Although there is limited evidence for a role of OCs in BD (Scott et al., 2006), length of gestation may play a role. A recent meta-analysis found that early (<37 weeks) or late gestational age (>39–42 weeks) increased risk of BD (Rodriguez et al., 2021), with one of the included studies finding a stronger risk for extreme prematurity (<32 weeks’ gestation) (Nosarti et al., 2012). As neither low nor high birth weight are associated with BD in meta-analyses (Scott et al., 2006), these findings point to an independent or confounding factor influencing pregnancy duration, rather than fetal growth (Rodriguez et al., 2021).

Concurrent examination of OCs and later development of psychiatric disorders was carried out in a small study (*n* = 333) which found that inadequate weight gain in mothers was positively associated with offspring development of schizophrenia spectrum disorders, but negatively associated with BD and major depression (Pugliese et al., 2019). Other distinctions between the two were that schizophrenia spectrum disorder was also positively associated with maternal stress, infections, and peripartum asphyxia, while BD was associated with a small head circumference (<32 cm) at birth.

The association between hypoxia-related OCs and SZ is greater in those with higher genetic risk (Mittal et al., 2008), and sibling studies have found a higher incidence of hypoxia-related OCs in SZ patients compared to their siblings without SZ (Cannon et al., 2000). Familial risk may also play a role in BD and OCs, as Singh et al. (2007) found that children of parents with BD may be at greater risk of OCs, compared to children of people without BD. Low birth weight was independently associated with increased risk of developing BD in offspring of parents with this disorder (Wals et al., 2003).

Methodological differences hinder inferences from the OC studies. Some examine the wide range of complications individually using definitions which often vary between studies, while others aggregate several OCs into a composite score. For individual OCs, the lack of standardization contributes to the inconsistent findings, and combining measures amplifies the problem in addition to introducing the possibility that inclusion of less harmful OCs in scores could dilute the overall effects.

One study reported a stronger association between a PRS derived from SZ-associated SNPs and SZ in those with OCs compared to those who did not experience OCs, with the most significantly associated SNPs mapping to genes highly expressed in placental tissue (Ursini et al., 2018). However, another study which applied this approach in five independent samples found no evidence that OCs interact with PRS or modulate the effect of the PRS on risk of SZ (Vassos et al., 2021). Therefore, there is currently insufficient evidence that OCs impact the association between SZ and genomic risk.

Several candidate genes have been investigated for interaction with OCs to modify risk of SZ, although reported interactions have rarely replicated (Schmidt-Kastner et al., 2012). However, a significant interaction was reported for three SNPs within AKT Serine/Threonine Kinase 1 (*AKT1*) (Nicodemus et al., 2008), one of which was subsequently replicated in a small study, but only in females (*n* = 67) (Joo et al., 2009). Future studies utilizing significant SNPs derived from SZ and BD GWAS may provide more comprehensive insights into G × E interactions for OCs.

### Infections

Increasing evidence points to the involvement of the immune system in the etiopathogenesis of psychiatric disorders. Several infectious agents have been associated with SZ, including viral, bacterial and parasitic infections (Arias et al., 2012), while for BD evidence is mixed (Benros et al., 2013; Barichello et al., 2016). When investigating broad groups of infections, factors such as exposure timing, virulence, strain of infection, and methods of assessing infections may lead to heterogeneity in findings. In terms of specific infections implicated in both SZ and BD, the strongest evidence is for *Toxoplasma gondii*, which has been associated with 25–50% increased odds of BD (Sutterland et al., 2015; de Barros et al., 2017), and 80% increased odds of SZ (Sutterland et al., 2015). *T. gondii* infection has also been associated with relevant biological processes, including increased dopamine production (Prandovszky et al., 2011), and pro-inflammatory factors related to mania and neuropathologic disorders (Hamdani et al., 2015). Behavioral alterations such as increased aggression and impulsivity (Cook et al., 2015) and increased risk of road traffic accidents (Gohardehi et al., 2018) have also been documented.

The timing of an infection may also be important, and pre- or perinatal elicitation of the immune response may be particularly deleterious during this early stage of development. In a seminal study of the 1957 Finnish influenza epidemic, pregnant women exposed to the virus during their second trimester had offspring with elevated risk of SZ (Mednick et al., 1988). Although findings regarding maternal infections during pregnancy have been mixed in meta-analyses, large register-based studies using clinical infection diagnoses support associations with SZ (Khandaker et al., 2012). Maternal bacterial infection during pregnancy was strongly associated with psychosis in offspring in a large United States study, with more severe effects for multisystemic (compared to localized) infections, and higher risk in males than females (Lee et al., 2020). Similarly, exposure to any bacterial infection in the first trimester increased risk of SZ in a Danish registry study (Sørensen et al., 2008).

A large meta-analysis found that viral central nervous system (CNS) infections in childhood were associated with 2.1× increased risk of adult SZ, but no significant increase was observed for bacterial CNS infections (Khandaker et al., 2012). These findings were substantiated by a large Swedish register study of over one million individuals which investigated the role of specific childhood CNS infections. No evidence supporting a role for any bacterial CNS infections was observed, but two serious viral infections, mumps and cytomegalovirus, were associated with subsequent psychoses (Dalman et al., 2008). Although the number of infections was greater in urban areas, adjustment for this did not influence the results.

For BD, a systematic review found mixed evidence for the role of 10 perinatal infections (Barichello et al., 2016). The strongest evidence was for *T. gondii* and cytomegalovirus (CMV) with positive reports in 5 of 9, and 5 of 11 studies, respectively. In a small sample from the Child Health and Development Study birth cohort, a strong association between maternal influenza infection and BD was reported using maternal medical records (Parboosing et al., 2013) and for BD with psychotic features using antibodies in maternal serum (Canetta et al., 2014). At present, evidence for a role of perinatal infections in BD is equivocal and investigations postnatally are also needed before firm conclusions can be drawn.

When examining infections later in life, infection timing needs to be considered to ascertain the direction of the effect, as individuals with SZ and BD generally have higher morbidity and lifestyle factors which increase susceptibility to infection (Regier et al., 1990; Brown et al., 1999). Other factors associated with increased likelihood of infections (e.g., stress, social inequality, and urban living) may also contribute (Marketon and Glaser, 2008; Semenza and Giesecke, 2008; Neiderud, 2015; Pini et al., 2019).

Due to the diverse range of infections associated with these disorders, a proposed mechanism underlying this effect is inflammation. Inflammatory processes can impact neuronal circuits, synaptic plasticity, reuptake of neurotransmitters (e.g., serotonin, noradrenalin, and dopamine), and stimulation of the hypothalamic–pituitary–adrenal (HPA) axis (Dantzer et al., 2008). A pro-inflammatory cytokine profile has been noted in SZ (Momtazmanesh et al., 2019) and BD patients (Barbosa et al., 2014). Defects in innate immunity may stem from genetic predisposition, or arise post-conception, or a combination of both. For instance, the “two-hit” hypothesis (Bayer et al., 1999), proposes that genetic or environmental factors in early life (first hit) disrupt the CNS and increase neurodevelopmental vulnerability to a ‘second hit’ later in life (Maynard et al., 2001). An environmental ‘hit’ could result from the direct mechanisms of a CNS infection or the indirect effects of systemic inflammation in response to any infection (Gardner et al., 2013; Benros and Mortensen, 2020). In large-scale epidemiologic studies, positive associations between autoimmune diseases and psychosis have been identified, suggesting that there may be common inflammatory components (Jeppesen and Benros, 2019).

A potential explanation for mixed results may be due to the influence of familial risk and shared genetic-environmental factors. Studies in Nordic countries which incorporated familial medical information have found no overall increased risk of psychosis in offspring following maternal infection during pregnancy (Clarke et al., 2009; Nielsen et al., 2013; Blomström et al., 2016). However, in mothers with a psychiatric disorder (indicating genetic predisposition) and infection during pregnancy there was increased psychosis risk for the offspring (Clarke et al., 2009; Blomström et al., 2016). Clarke et al. (2009) did not identify the same association for paternal psychiatric disorder, suggesting that it is not overall genetic liability, but rather the acute effects of infections during pregnancy in vulnerable mothers. These studies support possible G×E interactions between familial risk for psychiatric disorders and infections (Clarke et al., 2009; Nielsen et al., 2013; Blomström et al., 2016), but additional research is needed to explicitly test for this.

Genetic variation plays an essential role in the development and functioning of the immune system (Knight, 2013). Multiple lines of evidence support the role of immune genes in SZ (Ripke et al., 2013; Pouget, 2018), suggesting that genetic variation in immune response may underlie susceptibility to infections or aberrant response to them which, in turn, increase risk for SZ. Across several adult psychiatric disorders – including SZ and BD – risk variants have been identified within multiple immune-related pathways and processes (The Network and Pathway Analysis Subgroup of the Psychiatric Genomics Consortium, O’Dushlaine et al., 2015). A strong genetic correlation (*r*_g_ = 0.5) between susceptibility to infection and psychiatric disorders generally was also found in a GWAS performed in a large Danish cohort (*n* = 65,534), indicating shared genetic factors (Nudel et al., 2019). In support of this, variation in the MHC region, associated with susceptibility to infectious diseases (Matzaraki et al., 2017), has been repeatedly associated with SZ (Bergen et al., 2012; Mokhtari and Lachman, 2016; Ripke et al., 2020) and recently also with BD (Mullins et al., 2020). In another Danish study, SZ-PRS and a history of infections had independent effects on the risk for SZ (Benros et al., 2016), suggesting that an aggregated measure of genomic risk may inadequately capture the portion of genetic variants mediating risk through interaction with infectious agents.

There is also the possibility that individual SNPs may interact with specific infections to increase risk. A GWAS which aimed to identify gene variants that influence *T. gondii* seropositivity and their relationship with SZ risk did not find any genome-wide significant loci in two small samples, but there were suggestive associations for two schizophrenia-associated genes, *CNTNAP2* and *GABAR2* (Wang et al., 2019). They did not find a higher prevalence of *T. gondii* seropositivity in individuals with SZ compared to controls, which suggests that risk genes for SZ may also be involved in *T. gondii* susceptibility (rather than a causal relationship). A genome-wide interaction survey found an interaction between a SNP in the *CTNNA3* gene and maternal CMV infection increased risk of SZ (Børglum et al., 2014), and was subsequently replicated (Avramopoulos et al., 2015). Candidate gene studies have reported that maternal herpes simplex virus 2 (HSV-2) interactions with *GRIN2B* increase risk of SZ (Demontis et al., 2011), and for BD, toll-like receptor 2 (*TLR2*) polymorphisms reportedly interact with *T. gondii* infection to increase risk (Oliveira et al., 2016). However, independent replication would enhance the certainty of these candidate gene results.

Overall, these findings suggest that infections could have a direct or indirect role in triggering SZ and BD. Intriguingly, there may be some overlap in genetic liability between infections and psychiatric disorders, raising the possibility that immune-related genes mediating susceptibility or response to infections are indirectly risk genes for psychiatric disorders in a similar way as genes associated with nicotine dependence increase risk for lung cancer (Bierut, 2010).

### Season of Birth

Numerous studies have found a higher incidence of SZ in people born in the winter/spring months (Torrey et al., 1997; Karlsson et al., 2019), with increased risk at higher latitudes which are subject to greater seasonal variation (Davies et al., 2003). Although a general trend for excess winter/spring births has also been observed for BD, findings are fewer and less consistent than those for SZ (Torrey et al., 1997; Tsuchiya et al., 2003). In a systematic review, six out of nine studies supported an association between a winter/spring birth and risk of BD, but three other studies including one large study of 2.1 million individuals did not find evidence of an association (Tsuchiya et al., 2003).

Within the United Kingdom biobank samples, neither SZ-PRS nor SZ-associated CNVs were associated with season or month of birth, suggesting this is not directly genetically mediated (Escott-Price et al., 2019). However, it has long been hypothesized that seasonal variation in viral exposure, particularly influenza, may underlie the associations between SZ and births early in the year. Interactions between inflammation-related genetic variation in *HLA* genes (in the human MHC complex) and season of birth have been investigated in an attempt to support this hypothesis, with conflicting findings for *HLA-DR1* and winter birth in SZ (Narita et al., 2000; Tochigi et al., 2002). However, there was evidence of a significant interaction between *IL-4* (but not other cytokines) and season of birth on a milder diagnosis within the schizophrenia spectrum disorders, schizotypy (Alfimova et al., 2017).

For both SZ and BD, an interaction between the dopamine D4 receptor gene (*DRD4*) and season of birth has been reported (Chotai et al., 2003). Polymorphisms in *MTHFR, TPH1, SLC6A4* have also been investigated in terms of season of birth and SZ, but no significant interactions were identified (Chotai et al., 2003; Muntjewerff et al., 2011). Other potential candidate genes reportedly interacting with season of birth in terms of BD risk include tryptophan hydroxylase (*TPH1*) (Chotai et al., 2003), and the immune-related gene *HLA-G* (Debnath et al., 2013), but as with most of the candidate gene studies, these have yet to be replicated.

These genetic findings related to inflammatory genes are consistent with the possibility that the relationship between season of birth and SZ may be mediated by another environmental risk factor with seasonal fluctuations and immune-related processes. While infections have been most often implicated in this, pregnancy and birth complications, sunlight exposure, nutrition, temperature/weather or a combination of these are all viable possibilities.

### Migration

An increased risk of psychotic disorders, and SZ in particular, in migrants has been identified in several studies (Henssler et al., 2020). Some studies have found elevated risk even in 2^nd^ generation migrants, suggesting that the increased rate is not solely due to experiencing the stress of relocating itself, but of the accompanying social and environmental differences (Cantor-Graae and Selten, 2005). In contrast, elevated rates of BD have not been consistently shown among migrants (Swinnen and Selten, 2007) or their children (Cantor-Graae and Pedersen, 2013). It is possible the effects of migration may be related to specific clinical symptoms. For example, a large study using data from Swedish national registers found migrants had higher risk of psychotic disorders but lower risk of BD without psychosis, compared to Swedish-born individuals (Dykxhoorn et al., 2019).

There are many plausible, and potentially simultaneously occurring, factors which could contribute to increased risk in migrants, such as social factors (socioeconomic disadvantage, discrimination, and social isolation) (Davies et al., 2009), inadequate vitamin D levels (McGrath et al., 2010a), or vulnerability to infection (Rechel et al., 2013). One prominent theory is the social-defeat hypothesis, whereby social stressors, which can be more common among marginalized, vulnerable, socially-excluded or minority groups, may amplify an individual’s underlying risk for developing a psychiatric disorder (Selten et al., 2013). Risk may be related to having a visible minority status, as migrants from Africa moving to places such as Sweden, France, or Canada have highest risk of psychotic disorders (Tortelli et al., 2014; Anderson et al., 2015; Dykxhoorn et al., 2019). However, Finnish immigrants in Sweden have 2× greater incidence of SZ (Leão et al., 2006), indicating that increased risk is also evident for migrants without visible minority status. It has been proposed that patterns of risk may represent an underlying gradient of discrimination in a given country (McGrath, 2011), suggesting a substantial role for social environment on risk for psychosis. A range of other issues should also be considered, including factors related to differential pathways to care, diagnostic inaccuracies due to language and cultural differences, diagnostic bias, differences in genetic risk, and potential confounding due to socioeconomic factors.

No studies have investigated gene–environment interactions between migration and SZ or BD. Trans-ancestry genetic association studies for these disorders are still in nascent stages, but as these mature to more fully capture genetic risk for SZ and BD across populations, we will be better positioned to explore how genetic risk interacts with migration in the development of these disorders.

### Urbanicity

Urbanicity refers to the impact of residing in an urban area and is generally quantified based on population size or density. A meta-analysis of observational studies supported that there is increased risk of SZ for people living in urban areas compared to more rural areas (Vassos et al., 2012). There have been few studies investigating urbanicity in BD and results are mixed. For example in two large Danish studies, one supported an association for urbanicity at birth (Vassos et al., 2016), while the other did not (Mortensen et al., 2003). Urbanicity may be more strongly correlated with psychotic symptoms, and has been associated with higher rates of BD with psychosis, but not BD without psychosis (Kaymaz et al., 2006). Myriad possibilities have been suggested to mediate the association with urbanicity, such as the social (e.g., social capital, economic stress, or social fragmentation) or physical environment (e.g., air, noise, or light pollution) which require further investigation (Krabbendam and Van Os, 2005; Allardyce and Boydell, 2006).

Colodro-Conde et al. (2018) found that higher genetic risk for SZ was associated with urban living in four distinct samples from three countries. Using Mendelian randomization (MR), they concluded that those with increased genetic liability to SZ tend to reside in more densely populated urban areas and attributed this to selective migration. Similarly, data from the Danish registries also found that those with a higher PRS for SZ were more likely to live in the capital compared to more rural areas at age 15 (but not at birth), with attenuated, but still persisting, risks after adjustment for parental history of mental disorders (Paksarian et al., 2018).

In a large longitudinal cohort of children from the United Kingdom-based Avon Longitudinal Study of Parents and Children (ALSPAC) there was an association between SZ-PRS and neighborhood deprivation – but not necessarily more densely populated areas (Solmi et al., 2018). Similarly, an association between SZ-PRS and neighborhood deprivation was reported using data from Swedish registers, supporting the genetic selection theory that genetic risk for SZ predicts residence in deprived neighborhoods (Sariaslan et al., 2016). This indicates further work is necessary to examine the nuances of ‘urbanicity’ in greater depth, and the extent to which these associations are driven by genetic risk factors (Sariaslan et al., 2016).

Overall, these results suggest that underlying genetic risk may act in combination with urbanicity to increase risk of SZ, but perhaps not through a G×E interaction. More likely scenarios involve either active gene–environment correlation, in which people with increased genetic risk for SZ select an urban environment to live in, or a passive gene–environment correlation, whereby the association is driven by the genotype a child inherits from their parents who also determine their environment. Additional studies investigating these mechanisms are needed, as well as more comprehensive investigation of urbanicity and BD and the facets of urbanicity which may confer risk.

### Adverse Childhood Experiences

Childhood adversity has been extensively examined in the context of both BD and SZ with the majority of studies reporting positive associations (Matheson et al., 2013; Bailey et al., 2018; Rowland and Marwaha, 2018). The definition of ACEs across studies spans socioeconomic disadvantage, stressful life events, and childhood trauma. While definitions of stressful life events are highly heterogenous, common themes are: financial difficulties, parental separation, police involvement, neglect, physical/emotional abuse, or death of a family member. Although many studies use retrospective questionnaires, less subjective measures from registry data have also found increased risk (Wicks et al., 2005; Liang et al., 2016).

Childhood trauma is correlated with the severity of distinct symptoms in psychotic disorders, as well as an earlier age of onset in BD (Bailey et al., 2018; Rowland and Marwaha, 2018). Risks tend to be highest for more severe stressful events, such as the death of a first-degree relative, which are associated with developing both SZ (Liang et al., 2016) and BD with psychosis (Abel et al., 2014). Similarly, associations for BD may vary by type of trauma and BD type: BD1 was more prevalent in those who experienced sexual abuse, while experiencing emotional neglect has been associated with developing BD2 (Watson et al., 2014; Janiri et al., 2015).

Parental loss is associated with offspring psychosis irrespective of a family history of psychiatric disorders (Liang et al., 2016). However, a familial component may be evident for specific ACEs: offspring of suicide decedents are at greater risk for suicide (as well as psychotic disorders, drug disorders and violent criminal convictions) than offspring of living parents (Wilcox et al., 2010). Given that severe psychiatric disorders can influence parenting capacity (Ranning et al., 2015) and even children of parents with less severe mental illnesses are more likely to experience adversity (Pierce et al., 2020), it is often difficult to disentangle the factors contributing to psychiatric disorders.

The genetic and environmental effects of childhood trauma may act independently, as a small United Kingdom-based study did not find evidence of an interaction between SZ-PRS and retrospectively reported ACEs (Trotta et al., 2016). However, in individuals with high SZ-PRS, exposure to childhood adversity was associated with subtle psychosis expression, and positive and negative affect (Pries et al., 2020) which supports a possible G×E mechanism. To date, only one study of 402 BD cases has examined the effect of BD-PRS and childhood maltreatment on the clinical expression of BD (Aas et al., 2020). A lower BD-PRS was observed in individuals with BD diagnoses and more severe childhood maltreatment, particularly for emotional abuse, suggesting a possible additive relationship (Aas et al., 2020). There was a significant interaction between BD-PRS and childhood maltreatment with the risk of rapid cycling, but not with other clinical features.

There have also been a handful of G×E interaction studies for childhood trauma and psychiatric disorders involving candidate genes. Lower levels of brain derived neurotropic factor (BDNF), a neurotrophin which promotes growth and differentiation of neurons during brain development as well as synaptic plasticity and maintenance of neurons in adulthood, have been observed in SZ (Buckley et al., 2011). Interactions between childhood trauma and *BDNF* gene variants have been repeatedly shown in relation to BD and schizophrenia spectrum disorders (Mondelli et al., 2011; Aas et al., 2014; de Castro-Catala et al., 2016). Catechol-*O*-methyltransferase (*COMT*), which encodes an enzyme which catalyzes the breakdown of dopamine, has frequently been investigated in relation to ACEs and also cannabis use (Modinos et al., 2013). An interaction between childhood maltreatment and *COMT* was reported in the later development of psychosis (Vinkers et al., 2013), but this finding has not been consistently replicated (Trotta et al., 2019). There is also some evidence of interactions between early trauma and FKBP Prolyl Isomerase 5 (*FKBP5*) in mental disorders generally (Collip et al., 2013; Daskalakis and Binder, 2015). Larger studies of genetic risk with sufficient power to study distinct ACEs may provide further insight into potential G×E mechanisms.

### Cannabis Use

Cannabis use has been associated with increased risk of psychosis, a symptom of SZ and often BD, across several studies and populations (Marconi et al., 2016), and several longitudinal studies report that cannabis use is associated with subsequent development of SZ specifically (Arseneault et al., 2004). Few studies have examined the relationship between cannabis and BD, but one large longitudinal study found that weekly cannabis use was associated with 2.5× increased incidence of BD (Feingold et al., 2015).

Although cannabis use often co-occurs with the use of other substances, prolonged cannabis use remains associated with higher risk of psychotic symptoms after controlling for use of other drugs at baseline (Kuepper et al., 2011). Other substances, including cocaine/stimulant use have also been independently associated with SZ (Giordano et al., 2015). Associations across illicit drugs may be due to genetic predisposition, shared risk factors, or common neurological pathways (Agrawal et al., 2004; Khokhar et al., 2018). There are comparatively few studies examining the relationship between use of other illicit substances and psychosis; likely due to the reliance on self-reported data, lower prevalences, and the challenges in quantifying the exposure (i.e., potency, purity, and frequency of use). As cannabis is the most widely used recreational drug (WHO, 2018), understanding the mechanisms underlying its associations with psychosis has the greatest potential for harm reduction.

It has been suggested that cannabis use could be a cause or consequence of psychosis, or even that bidirectional relationships may exist. Giordano et al. (2015) found that cannabis abuse (defined using medical diagnoses and criminal convictions) 7 years prior to diagnosis of SZ was associated with 2× increased risk, with higher odds for cannabis use closer to diagnosis. Others have also found that in individuals with no history of psychotic experiences, cannabis use in adolescence precedes the onset of psychotic symptoms (Kuepper et al., 2011). However, some people developing psychosis may attempt to self-medicate with cannabis, even though evidence suggests this may exacerbate symptoms in patients with pre-existing psychiatric disorders. For example, continued cannabis use is associated with higher relapse rates in patients with psychosis (Schoeler et al., 2016), and with exacerbation of manic symptoms in those with pre-existing BD (Gibbs et al., 2015).

There is a significant genetic correlation between lifetime cannabis use and both SZ (*r*_g_ = 0.25) and BD (*r*_g_ = 0.29) implying some shared genetic etiology across common variants (Verweij et al., 2017; Pasman et al., 2018). Furthermore, in a sample of 2,082 healthy individuals, SZ-PRS was positively associated with self-reported cannabis use (Power et al., 2014), suggesting that individuals with greater genetic predisposition to SZ use cannabis more frequently. They corroborated this finding in a sample of twins: SZ-PRS burden was highest when both twins were users; intermediate with one twin user; and was lowest if neither twin reported cannabis use. MR has been used to formally discern the causal direction between cannabis use and SZ onset: one study found stronger evidence for a causal link from genetic liability to SZ to cannabis use (Gage et al., 2017), while the other reported the opposite (Vaucher et al., 2018). Additional studies with larger samples will be required before a consistent picture emerges. Overall, Convergent lines of evidence suggest some causal effects of cannabis use on developing SZ, along with a degree of genetic/familial confounding (Gillespie and Kendler, 2020).

French et al. (2015) investigated polygenic risk, cannabis use, and brain imaging measures in 1,577 individuals from three population-based adolescent and youth samples, and found that a higher SZ-PRS was associated with decreased cortical thickness, but only in males who used cannabis. As the age-related trajectories of gray and white matter differ between males and females during adolescence (Lenroot et al., 2007; Paus et al., 2010), sex differences in vulnerability to environmental factors over the course of brain development may also need to be considered in combination with genetic risk.

Cannabis use alone is not a sufficient trigger for psychosis in the general population; however, it could precipitate psychosis in susceptible individuals. Interactions between cannabis use and specific genes may help to explain the associations of cannabis with psychotic disorders. An interaction between cannabis and a SNP in the *AKT1* gene, which encodes a protein kinase in the dopamine signaling cascade, has been associated with a 2× greater risk of being diagnosed with a psychiatric disorder in both a sibling study (van Winkel, 2011) and a case-control study (Di Forti et al., 2012). A few studies have identified G×E interactions between cannabis use and variation in the *COMT* gene on psychosis (Caspi et al., 2005; Vinkers et al., 2013; Alemany et al., 2014), even though results have not always replicated (Zammit et al., 2011). Although these candidate genes are intuitively appealing due to their biological functions, examining the shared genetic etiology between cannabis use and both BD and SZ may be a more fruitful approach for uncovering specific genes conferring vulnerability.

## Emerging Risk Factors

The most robust environmental risk factors are the main focus of this review, but other exposures may emerge as key environmental factors in the future as evidence for them mounts. For example, air pollution is a well-established environmental health hazard and carcinogen, with adverse effects that extend beyond respiratory diseases and cancers (IARC Scientific, 2013), including neurological disorders such as Parkinson’s and Alzheimer’s disease (Shi et al., 2020). Furthermore, as proposed mechanisms for mediating the harmful effects of air pollution include oxidative stress, systemic inflammation, and neuroinflammation (Pope et al., 2004; Calderón-Garcidueñas et al., 2008), it is reasonable to speculate that there may be a link between air pollution and other disorders in which these processes are implicated such as SZ and BD. In Denmark, exposure to higher concentrations of ambient nitrogen oxide pollutants in childhood was associated with subsequent development of SZ and may account for some of the association with urbanicity (Antonsen et al., 2020). Similarly, in Sweden, neighborhood air pollution was positively associated with medications dispensed for psychiatric disorders in children and adolescents, although this was not evaluated specifically in relation to SZ or BD (Oudin et al., 2016). In China, even short-term exposure to ambient air pollution has been associated with increased daily outpatient visits for SZ in adults with and without prior SZ diagnoses (Liang et al., 2019). A recent study evaluating risk from different types of environmental pollutants was conducted for 151 million individuals in the United States, and 1.4 million individuals in Denmark (Khan et al., 2019). Taking into account urbanicity and socioeconomic status, they found correlations between air pollution and BD in both countries and also with SZ in Denmark. Although it is the largest and most thorough study on air pollution to date, additional evidence is needed before it can be conclusively established as a risk factor for either disorder.

Additional research is also warranted examining the complex and variable components of ambient air pollutants, as well as indoor air pollutants such as fuels burned for cooking. Cigarette smoke is an air pollutant with abundant evidence of negative health effects, and these include some reports of strong associations with SZ and BD (De Leon and Diaz, 2005; Jackson et al., 2015). The effects are not limited to inhalation of chemicals in tobacco smoke, as there is also evidence for a modest association between non-affective psychosis and ‘snus’ (a smokeless tobacco product) (Munafò et al., 2016). The association has generally been attributed to self-medication and reverse causation (Quigley and MacCabe, 2019). Although this may be true for some individuals, it cannot fully explain the association, and there are a number of alternative biological and genetic explanations (Quigley and MacCabe, 2019). Evidence supports a shared genetic liability between both smoking and psychosis (Kendler et al., 2015), as well as correlation between genetic variants for SZ and nicotine dependence (Quach et al., 2020). MR analyses also suggest a causal relationship between smoking and both SZ and BD (Vermeulen et al., 2019; Wootton et al., 2020). The growing literature in this area suggests that nicotine/smoking will soon gain greater recognition as a contributing risk factor for these disorders.

Traumatic brain injury (TBI) is reported to precede the onset of a range of psychiatric conditions (Bryant et al., 2010), including SZ and BD (Molloy et al., 2011; Orlovska et al., 2014), although it is difficult to discern whether TBI causes psychiatric symptoms or vice versa. Risk for TBI is greater in individuals with a genetic predisposition to psychosis suggesting possible genetic correlation (Molloy et al., 2011). In siblings discordant for TBI before age 25, siblings who experienced TBI had twice the risk of subsequent psychiatric inpatient hospitalization (Molloy et al., 2011). However, it is not known whether they had higher genetic risk for psychiatric disorders than their siblings without these disorders or TBI. Since children and adolescents who experience TBI have elevated risks of a wide range of medical and social problems in later life (Molloy et al., 2011), further research is required to determine the mechanisms by which these injuries contribute to subsequent problems, including the development of SZ and BD.

Hearing impairment in childhood has been reported as a risk factor for developing psychosis in adolescence (van der Werf et al., 2010), and later life (Linszen et al., 2016), although there was no evidence for an interaction with SZ-PRS (Guloksuz et al., 2019). A connection between hearing impairment and BD has not yet been examined, and the overall paucity of studies on this topic makes it difficult to evaluate the validity of this potential relationship at this time.

There is growing interest in nutritional medicine in psychiatry and the relationship between nutrient deficiencies and psychiatric disorders (Sarris et al., 2015). For example, folate is an essential nutrient obtained from the diet, and there is evidence of lower serum folate levels in individuals with BD and SZ (Wang et al., 2016; Hsieh et al., 2019). Polymorphisms in the methylenetetrahydrofolate reductase (*MTHFR*) gene, a key enzyme in the folate cycle, may be partly driving this association in SZ (Lewis et al., 2005; Yadav et al., 2016) and BD (Peerbooms et al., 2011). Another example is Vitamin D, a neurosteroid essential for brain development and function, which is produced when skin is exposed to sunlight and acquired through dietary sources (Cui et al., 2021). Low levels of vitamin D are well-documented in SZ (Valipour et al., 2014), but only occasionally reported in BD (Cereda et al., 2020). Two studies report an association between neonatal vitamin D deficiency and increased risk of SZ (McGrath et al., 2010b; Eyles et al., 2018). This observation has been theorized to underlie the associations between winter/spring births and SZ, since maternal sun exposure would likely be low in the months preceding birth. Vitamin D may also be relevant as a mediator of the migration association with SZ, particularly for dark-skinned individuals moving from regions with high sun exposure to less sunny areas (Eyles et al., 2013). However, definitive associations with developing SZ have not yet been shown, and little research has been conducted for BD. Furthermore, most studies on nutrient deficiencies have been cross-sectional and additional prospective studies are required to assess potential causal relationships with SZ or BD.

## Summary and Future Directions

Several environmental exposures have been investigated in terms of SZ and BD risk, and there is moderate evidence that ACEs and certain types of infections are risk factors for both. For winter/spring birth, OCs, migration, urbanicity, and cannabis use, however, more robust associations have only been identified for SZ. Undoubtedly, the research for BD lags behind the larger body of work for SZ, and it remains unclear whether the reported differences in environmental risk between BD and SZ are due to sample size and methodological differences, or true etiological distinctions. For both disorders, evidence implicating other exposures such as air pollution and nicotine/smoking is still growing, while still other exposures may yet be discovered. Only a small fraction of environmental variables that individuals are exposed to have been investigated, and, in time, more environmental risk factors for SZ and BD will likely come to light.

One possibility for inconsistent associations, particularly for BD, is that some risk factors may be related to specific disease types or symptoms. For instance, while the evidence for urban environmental risk is stronger for SZ (Krabbendam and Van Os, 2005) than for BD in general, there was an association between urban residence and BD1 but not BD2 (Kaymaz et al., 2006) which suggests that urbanicity could be conferring risk for symptoms that are common across SZ and BD1. Similarly, childhood trauma has been associated with increased risk for both SZ and BD, with evidence suggesting a higher prevalence of BD1 in those who experienced sexual abuse, while BD2 is more prevalent following emotional neglect (Watson et al., 2014; Janiri et al., 2015). Similarly, several studies across multiple environmental domains, but most notably for cannabis use, examined psychosis as an outcome. Since psychosis is much more common in BD1 than BD2 (and a defining characteristic of SZ), this also has stratifying effects. Therefore, studies which pool together psychosis, BD types, and types of ACEs may dilute or miss associations.

Sex differences in the prevalence and manifestation of SZ and BD have been widely investigated, but rarely with regard to environmental risk. For some exposures, the rates may differ between the sexes. For example, there is higher reported cannabis use (Hasin et al., 2015) and increased susceptibility to infections in early life in males (WHO, 2007). The effect of an exposure can also differ between the sexes. Despite a lower prevalence of cannabis use disorder, females who do have this disorder have increased risk of BD compared to males (Khan et al., 2013). Furthermore, the immune response to infections differs between the sexes (van Lunzen and Altfeld, 2014), which has ramifications for how these processes may mediate risk for SZ and BD. It is also well documented that male sex is associated with worse pre- and perinatal outcomes (Di Renzo et al., 2007). However, there is mixed evidence as to whether OCs confer higher risk of SZ in males compared to females (Verdoux et al., 1997; Dalman et al., 1999), or just result in earlier age of onset in males (Kirov et al., 1996). As larger studies of environmental measures impacting SZ and BD are conducted, separate estimations of their effects on men and women should be assessed.

**FIGURE 2:**
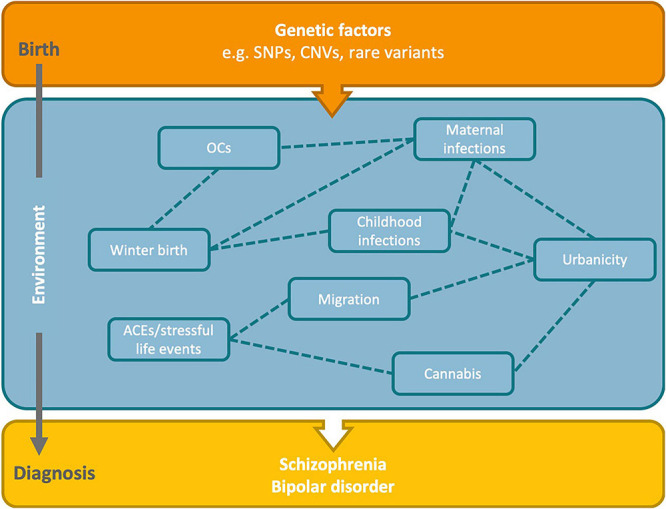
Hypothesized relationships between risk factors and genetics in the development of schizophrenia and bipolar disorder. Genetic factors present from birth may interact with environmental factors across the life course to increase the risk of SZ and BD. There may also be complex interrelationships between the environmental risk factors. Dashed lines indicate potential relationships between the identified environmental risk factors. OCs, obstetric complications; ACEs, adverse childhood experiences; SNPs, single nucleotide polymorphisms; CNVs, copy number variants.

Complex disorders arise due to intertwined genetic and environmental influences, and myriad constellations of risk are possible (Figure 2). For instance, people who experience childhood trauma are more likely to compound their risk by using cannabis (Harley et al., 2010). For winter/spring birth, infections, and urbanicity there are several overlapping aspects: there are seasonal fluctuations in most communicable diseases, and there is greater spread of infections in more densely populated urban areas. Then there is the consideration that the risk-increasing effects of urbanicity for psychosis are greater for those with familial risk of psychotic disorder (Van Os et al., 2003). Therefore, familial risk and several environmental factors could have additive or synergistic effects on increasing the risk for psychiatric disorders. To dissect these more complex, multifactorial relationships requires specialized statistical methods, such as structural equation modeling, as well as large, well-characterized cohorts.

Environmental risk factors are not acting in isolation, and investigations of genetic context as a modifying factor have evolved over time. Studies incorporating both genetic data and environmental risk have historically been hindered by insufficient knowledge of the genetic markers conferring risk for SZ and BD. Several candidate genes thought to confer risk for BD or SZ, but demonstrating inconsistent associations, have been investigated for some exposures in G×E interaction studies (Table 1). For example, *COMT* is reported to interact with both cannabis and ACEs in SZ (Vinkers et al., 2013), while *BDNF* interactions with ACEs have been associated with both BD and SZ (Mondelli et al., 2011; Aas et al., 2014; de Castro-Catala et al., 2016). As with nearly all candidate gene association studies prior to the GWAS era, these have rarely been replicated.

In comparison to environmental data, harmonization is more attainable for genetic data which are fixed over time within individuals and are more objectively quantifiable. Now that GWAS using samples amalgamated across several studies have revealed numerous loci with high confidence associations, these loci can be tested for interactions with environmental exposures individually or in aggregate (as PRS). Although much progress has been made in identifying the genetic risk factors for both SZ and BD, it is certain that additional genetic associations remain to be discovered. The mechanisms of action linking genetic markers to the behavioral changes characterizing these disorders also require further investigation.

Power has been another key limitation of most prior G×E research. Power calculations using sample sizes from previous G × E studies suggest that some of the reported results may represent false positives (Duncan and Keller, 2011). As larger samples become available, well-powered interaction studies will become feasible.

Some systematic changes to improve this field of research are also warranted. Sampling bias is a common issue in epidemiological research, and this area is no exception. For example, individuals with higher SZ-PRS are more prone to drop out of studies, leading these individuals to be underrepresented and risk underestimated (Martin et al., 2016). Study designs that are robust to this, such as nationally representative register-based studies, could be used more often. There is growing recognition that large electronic health record databases, and in particular, national registers from Nordic countries which hold rich, high-quality data, familial relationships, and cover the entire population of a country, are excellent resources for use in psychiatric research (Allebeck, 2009). Another crucial bias to address is the strong overrepresentation of people of European ancestry in genetic research. Not only does this hinder application of PRS beyond the study population from which the GWAS was conducted, but the lack of diversity may also exacerbate existing health disparities (Martin et al., 2019). Psychiatric GWAS are increasingly incorporating a broader range of populations (Fiorica and Wheeler, 2019; Lam et al., 2019; Bigdeli et al., 2020), but more samples representing global diversity are needed.

In addition to elucidating the developmental origins of SZ and BD, G×E interactions may partially explain the heritability gap between twin studies and molecular heritability estimates. Genetic variants discovered by GWAS so far account for a smaller proportion of variance than was estimated in twin studies, with heritability estimates of around 75 and 80% from twin studies (Smoller and Finn, 2003; Sullivan et al., 2003), and 25 and 22% SNP heritability for BD and SZ, respectively (Lee et al., 2013). One plausible explanation is that GWAS are incompletely capturing the large portion of risk from common variants with small effect sizes, and entirely missing copy number variants and other rare genetic variants (Manolio et al., 2009). Overestimation of heritability in twin studies due to epistatic or epigenetic factors is also possible (Trerotola et al., 2015). However, a key distinction in family studies vs. individuals from the general population is not only the shared genetic components, but also the considerable sharing of environmental exposures.

The high degree of shared genetic risk between psychiatric disorders (Bulik-Sullivan et al., 2015) raises the possibility that environmental exposures could be determining factors for the specific diagnoses that emerge. Few studies have attempted to test this for SZ and BD by concurrently studying environmental risk factors for these disorders, and large-scale, parallel investigations of environmental risk are needed. Studies included in this review used designs ranging from longitudinal register-based population cohorts to cross-sectional studies with retrospective assessments of exposures. The resulting heterogeneity in the sample characteristics, psychiatric outcomes, exposure timings and measurements has hindered interpretation of environmental contributions to risk for BD and SZ. Concurrent examination of BD and SZ will facilitate comparisons of how environmental exposures act independently and in conjunction with genetic risk to shape these diagnoses.

In summary, although some environmental risk factors have been identified for SZ, few have been with certainty for BD, and the extent to which these are shared remains largely unknown. For both disorders, interactions between environmental and genetic risk factors are also not well understood and merit further investigation. Elucidating the mechanisms which give rise to these related conditions may reveal opportunities for prevention efforts and identify therapeutic targets. Further research is needed to understand how environmental and genetic risk factors exert their influences biologically to shape an individual’s propensity for these disorders.

## Author Contributions

NR was responsible for the literature search, producing the figures, and drafting and refining the manuscript. SB provided supervision, critical feedback, and revision of the manuscript. Both authors contributed to manuscript preparation and have read and approved the submitted version.

## Conflict of Interest

The authors declare that the research was conducted in the absence of any commercial or financial relationships that could be construed as a potential conflict of interest. The handling editor declared a past co-authorship with one of the authors SB.
